# Obstructive sleep apnea presenting as pseudopheochromocytoma

**DOI:** 10.11604/pamj.2016.23.75.8979

**Published:** 2016-03-10

**Authors:** Hela Marmouch, Sondes Arfa, Sameh Graja, Tensim Slim, Ines Khochtali

**Affiliations:** 1 Department of Endocrinology and Internal Medecine, Fattouma Bourguiba University Hospital, Monastir, Tunisia

**Keywords:** Obstructive sleep apnea, hypertension, catecholamines, pheochromocytoma

## Abstract

A 52-year-old female with a history of poorly controlled resistant hypertension was admitted to our hospital with severe hypertension. She had a history of fatigue and intermittent episodes of palpitations. Laboratory evaluation was significant for elevated 24-h urinary catecholamine levels (3,5 times the upper normal levels). This case was presenting with a clinical and biochemical picture indistinguishable from that of pheochromocytoma. However, neither computed tomography nor meta-iodo-benzyl-guanidine scintigraphy detected any catecholamine-producing tumor in or outside the adrenal glands. Our patient was screened with full polysomnography because of heavy snoring, daytime somnolence and obesity. It revealed severe obstructive sleep apnea syndrome. After three months of continuous positive airway pressure therapy, the patient experienced resolution of his presenting symptoms, improved blood pressure control and normalization of his urinary catecholamine levels. This case highlights sleep disordered breathing as a potentially reversible cause of pseudo-pheochromocytoma.

## Introduction

The obstructive sleep apnea syndrome (OSAS) has a well-documented association with increase cardiovascular morbidity and mortality [1]. The patients with OSAS have a high prevalence of hypertension (HTA) and the OSAS may present similar to a pseudo-pheochromocytoma. Indeed, this syndrome can elevate catecholamine secretion and increased sympathetic activity which may mimic the biochemical profile of pheochromocytoma [2]. Treatment of OSAS may normalize the effects of this sympathetic overdrive and resolve excessive catecholamine secretion. In this article, we report a case with pseudo-pheochromocytoma caused by OSAS; a common medical condition which is less recognized as a cause of raised catecholamines.

## Patient and observation

A 52-year-old female was admitted in the department of endocrinology with severe headache, dizziness, and visual problems. She had been treated for hypertension diagnosed eight months ago. She was taking Acebutolol 200 mg /day, amlodipine 10 mg/day, and a combination of angiotensin-conversion enzyme inhibitor (Captopril 300 mg daily), Indapamide 1,5 mg daily but experienced several hypertension peaks and hypotension. She adhered to treatment, was neither using traditional herbal medication nor illicit drugs, had no known family history of endocrine disorders and she did not smoke. At admission, his blood pressure was 200/120 mm of Hg. His body mass index was 31 kg/m^2^. Physical examination was unremarkable. Fundoscopic examination of the eye revealed hypertensive retinopathy stage 2 of Kirkendall classification. Electrocardiography and echocardiography found slight left ventricular hypertrophy with normal cardiac output. Laboratory tests found an hypokalemia= 3,5 mmol/l, an elevated creatinine level at 120µmol/l with normal fasting glycemia and lipid profile. Laboratory evaluation was significant for elevated 24-h urinary catecholamine levels (3,5 times the upper normal levels). Ultrasound and Computed Tomography-scan showed kidneys with normal size, good cortical index, and normal vessels. In addition, neither computed tomography nor meta-iodo-benzyl-guanidine (MIBG) scintigraphy detected any catecholamine-producing tumor in or outside the adrenal glands. On further questioning, she admitted to a long history of loud snoring and day time somnolence. His Epworth sleepiness score (ESS) was 16, suggesting excessive daytime sleepiness (normal <10). Subsequently, a home-based overnight polysomnography showed evidence of severe OSA with an apnoea hypopnoea index (AHI) of 30 events/hour (normal <5). We stopped slowly Acebutolol and Indapamide, continued the angiotensin-conversion enzyme inhibitor and Calcium channel blockers and added Prazosine 5 mg daily to patient′s treatment. Following 3 weeks of overnight continuous positive airway pressure (CPAP) therapy, the patient recorded daily self-measured blood pressure at home with a semi-automatic sphygmomanometer. All blood pressure measures were under 130/80 mm of Hg like the one measured during consultation. Three months later, a new fundoscopic eye examination realized, showed regression of retinopathy to stage 1 and his urinary cathecolamine levels normalized. She had felt better without any symptom, we reduced captopril to 200 mg /day and stopped prazosine and amlodipine to prevent hypotension episodes and she had a stable blood pressure within normal ranges even after exercise.

## Discussion

OSAS is characterized by upper airway occlusion and recurrent cessation of respiratory flow during sleep. Hypoxia ensues, followed by repeated arousal episodes in an attempt to restore airway patency. Patients complain of disturbed sleep and daytime somnolence, and relatives may give a long history of loud snoring. Diagnosis is made by overnight monitoring of arterial oxygenation, chest wall movement, and airflow, proceeding to full polysomnography in equivocal cases. This syndrome has a well-documented association with increased cardiovascular morbidity and mortality [1] and has been shown to be an independent risk factor for the development of systemic hypertension [3]. Since the 1980s it has been thought that sympathetic activity is up-regulated in patients with sleep-disordered breathing. Twenty-four-hour urinary catecholamine levels were initially found to be increased in patients with untreated sleep apnea compared with those with narcolepsy [1, 4]. Evidence is accumulating to suggest a role for sympathetic over activity in the pathophysiology of these observations [5]. Indeed a small cohort of male patients with severe sleep apnea and elevated urinary catecholamine levels was found to lose the normal diurnal variation in sympathetic excretion, suggesting increased nocturnal sympathetic activity. OSAS, however, is not the only cause of pseudo-pheochromocytoma as they could be showed in other causes, notably physiological stress, antipsychotic drugs [6], anti-parkinson drugs, tricyclic antidepressants and cocaine use. Panic attack, alcohol withdrawal and carcinoid syndromes are other situations that can explain pseudo-pheochromocytoma. None of these conditions were found in our patient who was previously healthy without any medication and denied any alcohol or illicit drug abuse. Other causes of secondary hypertension such as primary hyperaldosteronism, hyperthyroidism, hypercorticism, and endocrine neoplasms were excluded by hormonal explorations. In addition, mechanisms by which chemoreflex dysfunction may contribute to chronically elevated sympathetic tone and ultimately hypertension are discussed [7]. In one series, Hoy et al described 5 patients in whom no tumors were identified, and primary treatment for OSA normalized both clinical features and catecholamine excess [8]. These and similar reports strengthen the link between OSA and elevated sympathetic activity. While the exact mechanism of this association is unknown, hypoxia-induced endothelial dysfunction and oxidative stresses are thought to contribute. Further, repetitive arousals in response to anoxic/hypoxic episodes can lead to increased urinary catecholamine and metanephrine levels [9]. Nasal CPAP therapy can reduce this increased sympathetic tone by preventing upper airway closure and mitigating repetitive arousals, as evidenced by normalization of sympathetic markers following treatment. Further, CPAP has been shown to decrease parameters of sympathetic tone and decrease day and nighttime blood pressure recordings [9]. Nasal CPAP therapy is recommended not only to improve hypertension and catecholamine hypersecretion but also to distinguish the condition from pheochromocytoma. Finally this case represents a patient with undiscovered OSAS and difficult to control hypertension, with clinical and biochemical evidence of increased sympathetic activity mimicking a pheochromocytoma. When imaging fails to reveal the presence of a catecholamine secreting tumor, a diagnosis of OSAS induced pseudo-pheochromocytoma should be considered. Recognizing this association is important, as primary treatment for OSAS may lead to a resolution of symptoms and normalization of urinary catecholamine and metanephrine levels.

## Conclusion

Finally this case represents a patient with untreated OSAS and difficult to control HTA, with clinical and biochemical evidence of increased sympathetic activity mimicking a pheochromocytoma. Nasal CPAP therapy allows improvement of hypertension with catecholamine hypersecretion and distinction of this condition from pheochromocytoma. We suggest that a diagnosis of OSAS should be considered in patients with clinical and biochemical evidence of catecholamine excess in whom a catecholamine producing tumor cannot be identified. Recognizing this association is important, as primary treatment for OSAS may lead to a resolution of symptoms and normalization of urinary catecholamine and metanephrine levels.
